# Annual Wellness Visits and Early Dementia Diagnosis Among Medicare Beneficiaries

**DOI:** 10.1001/jamanetworkopen.2024.37247

**Published:** 2024-10-08

**Authors:** Huey-Ming Tzeng, Mukaila A. Raji, Yong Shan, Peter Cram, Yong-Fang Kuo

**Affiliations:** 1 School of Nursing, University of Texas Medical Branch, Galveston; 2 Sealy Center on Aging, University of Texas Medical Branch, Galveston; 3 Department of Internal Medicine–Geriatrics & Palliative Medicine Division, School of Medicine, University of Texas Medical Branch, Galveston; 4 Office of Biostatics, University of Texas Medical Branch, Galveston; 5 Department of Biostatistics and Data Science, School of Public and Population Health, University of Texas Medical Branch, Galveston; 6 Department of Internal Medicine, School of Medicine, University of Texas Medical Branch, Galveston; 7 Department of Internal Medicine, Faculty of Medicine, University of Toronto, Toronto, Ontario, Canada

## Abstract

**Importance:**

Early recognition of cognitive impairment is key to optimal dementia care. No previous research has examined the probability of developing mild cognitive impairment (MCI) or Alzheimer disease and related dementias (ADRD) at 5-year follow-up among older adult Medicare beneficiaries by receipt of an annual wellness visit (AWV).

**Objective:**

To assess the association of incident AWV with the first ADRD or MCI diagnosis among older adults with Medicare fee-for-service benefits.

**Design, Setting, and Participants:**

This retrospective population-based cohort study used 100% Texas fee-for-service Medicare data from 2015 to 2022. Participants comprised 549 516 community-dwelling Medicare beneficiaries aged 68 years or older in 2018, with complete Medicare fee-for-service Parts A and B and no Medicare Advantage plan enrollment for 2015 to 2018.

**Exposure:**

Medicare AWVs.

**Main Outcomes and Measures:**

The first MCI or ADRD diagnosis (reported as MCI or ADRD diagnosis, MCI diagnosis, and ADRD diagnosis) from the AWV index date in 2018 through December 31, 2022.

**Results:**

In this cohort study of 549 516 Medicare beneficiaries with no diagnosis of MCI or ADRD in 2015 to 2017 (mean [SD] age, 76.7 [6.6] years; 289 932 women [52.8%]), 66 433 (12.1%) had an incident AWV in 2018. Annual wellness visit recipients were more likely than those who did not receive an AWV to be female, to be non-Hispanic White (followed by Hispanic, non-Hispanic Black, and other), to have more education, to reside in a metropolitan area, to have more comorbidities, and to have a primary care professional in the 12 months before the AWV index date. After propensity score matching, AWV receipt was associated with a 21% increase in MCI diagnosis (hazard ratio, 1.21 [95% CI, 1.16-1.27]) and a 4% increase in ADRD diagnosis (hazard ratio, 1.04 [95% CI, 1.02-1.06]). The increase in MCI diagnosis associated with AWV was larger when the AWV was censored or treated as a time-dependent covariate in the follow-up period.

**Conclusions and Relevance:**

These findings indicate that AWV recipients had a timelier first MCI diagnosis than those who did not receive an AWV, but first ADRD diagnosis differed little. This study suggests that the Medicare AWV health policy may increase MCI identification, prompting more specialized care.

## Key Points

**Question:**

What is the association of an incident annual wellness visit (AWV) with the first diagnosis of mild cognitive impairment (MCI) or Alzheimer disease and related dementias (ADRD) among older adults with Medicare fee-for-service benefits?

**Findings:**

In this cohort study of 549 516 Medicare beneficiaries, AWV receipt was associated with a 21% increase in MCI diagnosis and a 4% increase in ADRD diagnosis compared with no AWV. The increase in first MCI diagnosis associated with AWV was larger in 2 sensitivity analyses.

**Meaning:**

This study suggests that Medicare AWVs may increase early MCI recognition, leading to more proactive care in such older adults.

## Teaser

This cohort study assesses the association of an incident annual wellness visit with the first diagnosis of mild cognitive impairment and Alzheimer disease and related dementias among older adults with Medicare fee-for-service benefits.

## Introduction

Optimal dementia care depends on early recognition of cognitive impairment and timely diagnosis of Alzheimer disease and related dementias (ADRD).^1,2,3,4,5^ Optimal dementia care should reflect what matters most to patients and adhere to evidence-informed ADRD stage-specific health care.^6,7,8,9,10,11,12,13,14,15^ Early recognition of cognitive impairment could prompt clinicians to initiate conversations about advance care planning and assess patients for potentially treatable factors associated with cognitive impairment (eg, hypothyroidism, poor hearing, and sleep apnea).^5,16,17,18^ Early diagnosis of cognitive impairment can reduce exposure to potentially inappropriate medications,^19^ reduce acute care use,^20,21,22,23^ decrease falls,^24,25,26,27,28,29,30^ and increase adoption of advance care planning.^31,32^ Only 8% of Medicare beneficiaries with mild cognitive impairment (MCI) receive an MCI diagnosis in primary care clinics.^33^ Patient access to dementia assessment often limits timely diagnosis of cognitive impairment.^15^

One way to improve early cognitive impairment recognition^15^ is through annual wellness visits (AWVs), which typically include cognition and fall risk assessment, medication reconciliation, advance care planning consultation, and personalized prevention plans.^34,35,36,37^ The Centers for Medicare & Medicaid Services began reimbursing for AWVs in 2011 without cost to Medicare enrollees aged 65 years or older.^38^ Annual wellness visit rates increased from 7.9% in 2011 to 21.9% in 2015.^39^ In 2017, 32.2% of all Texas fee-for-service Medicare beneficiaries received AWVs.^30^ Disparities in AWV use include fewer visits for men, non-White beneficiaries, and rural beneficiaries.^30^

In 2020, the US Preventive Services Task Force^40^ recommended against routine cognitive screening, a major component of the AWV program. However, a systematic review and meta-analysis of 17 chronic heart failure studies (mean age, 75.6 years) and 14 chronic obstructive pulmonary disease studies (mean age, 66.3 years) found an overall prevalence of any cognitive impairment of 32% in patients with chronic obstructive pulmonary disease and 31% in those with chronic heart failure.^41^ Another study found a 46% prevalence of MCI in older adults attending an ophthalmology clinic.^42^ Tailoring therapy to cognitive impairment status may reduce treatment nonadherence, medication errors, emergency department or hospital visits, and missed appointments, preventable events in patients with chronic conditions and unrecognized cognitive impairment.^42^

To our knowledge, no study has examined the rate of developing a first MCI or ADRD diagnosis at a 5-year follow-up for an AWV in Medicare enrollees. In this cohort study, we assessed the association of incident AWV in 2018 with the first ADRD or MCI diagnosis from the AWV index date through the end of 2022 in Medicare fee-for-service beneficiaries in Texas. We hypothesized that beneficiaries with an AWV would have an increased rate of first MCI diagnosis and no difference in first ADRD diagnosis, compared with those without an AWV.

We focused on Texas, a state with the largest proportion of Hispanic and Black individuals of all US states.^43,44,45^ Texas also has a high rate of individuals with dementia^43,44,45,46,47^ and one of the highest population estimates of those with ADRD.^47^ In 2022, the Alzheimer disease mortality rate in Texas was 38.8% per 100 000, the ninth highest rate among states.^48^ Texas also has the sixth worst overall ranking in health system performance.^49^ Texas has the largest rural population in the US^50,51^; rural residents are at risk for poor health due to limited health care access.^52^ Last, AWV adoption rates vary by clinic types and region; nationally, San Angelo, Texas, has the lowest regional AWV receipt rate.^53^ In short, residents of Texas have a high cognitive impairment risk and low AWV use.

## Methods

### Data Source

This retrospective cohort study used enrollment and claims data from 100% of Texas Medicare administrative data from 2015 to 2022, including Medicare Beneficiary Summary files, Medicare Provider Analysis and Review files, outpatient claims, and carrier files. The University of Texas Medical Branch institutional review board approved this study. Consent was waived because the data were deidentified. We followed the Strengthening the Reporting of Observational Studies in Epidemiology (STROBE) reporting guideline.^54^

### Cohort

The study population was community-dwelling Medicare beneficiaries aged 68 years or older in 2018. We restricted analysis to fee-for-service beneficiaries with complete Medicare Parts A and B and no Medicare Advantage plan enrollment for all of 2015 to 2018. We excluded beneficiaries with a previous diagnosis of ADRD or MCI or an AWV based on 2015-2017 claims (eTable 1 in Supplement 1).

For beneficiaries with an AWV visit in 2018, the first AWV date in 2018 was the index date. For beneficiaries with no AWV visit in 2018, we randomly assigned each an index month in 2018, matching the frequency of index months of the AWV cohort, and the 15th of the month as their index day. After assigning the AWV index date, we excluded any beneficiaries with AWV, ADRD, or MCI, as well as those in long-term nursing homes before the 2018 AWV index date. We kept the cohort with complete data in our analysis (Table 1).

**Table 1.** Cohort Derivation Steps

Selection criterion	No. (% of the previous step)
1. Total Medicare fee-for-service beneficiaries residing in Texas in 2018	4 228 611
2. Removed Medicare beneficiaries who were not ≥68 y in 2018	2 874 305 (68.0)
3. Restricted the Medicare beneficiaries with complete Medicare A and B and no Medicare Advantage (also called health maintenance organization) enrollment during 2015-2018	1 185 645 (41.2)
4. Removed beneficiaries with a cognitive impairment–related diagnosis (ie, mild cognitive impairment or Alzheimer disease and related dementias) in 2015-2017	1 035 665 (87.4)
5. Excluded beneficiaries lacking a valid sex signifier	1 035 665 (100.0)
6. Excluded long-term nursing home residents in 2018	1 030 694 (99.5)
7. Excluded beneficiaries lacking residency information (ie, the first 3 digits of the zip code of the resident address)	1 003 095 (97.3)
8. Excluded beneficiaries having any annual wellness visit in 2015-2017	578 605 (57.7)
9. Excluded beneficiaries without educational information in their residential areas	559 471 (96.7)
10. Excluded beneficiaries without the physical inactivity or association rates in their residential areas	549 516 (98.2)

### Measures

Our primary exposure was an AWV in 2018. Medicare allows 1 AWV every 12 months for beneficiaries with Medicare Part B medical insurance for more than 12 months and with no initial preventive physical examination or AWV providing a personalized prevention plan within the preceding 12 months. Beneficiaries have no copayment for an AWV (eTable 1 in Supplement 1).^55^

### Outcome

Our outcome was the first MCI or ADRD diagnosis from the AWV index date through the end of 2022. We reported the findings in 3 ways: MCI or ADRD diagnosis, MCI diagnosis, and ADRD diagnosis.

We censored patients at the end of the study (December 31, 2022), loss of Medicare insurance (Parts A and B), or switch to a Medicare Advantage plan, whichever happened first. We treated death as a competing risk in all analyses. For MCI or ADRD analysis, we censored the other diagnosis. Alzheimer disease and related dementias diagnosis was based on *International Classification of Diseases, Ninth Revision, Clinical Modification* (*ICD-9-CM*) and *International Statistical Classification of Diseases, Tenth Revision, Clinical Modification* (*ICD-10-CM*) codes from inpatient and outpatient claims listed in the Centers for Medicare & Medicaid Services Chronic Conditions Data Warehouse algorithm.^56^ We identified MCI diagnosis using *ICD-9-CM* code 311.83 and *ICD-10-CM* code G31.84 (eTable 1 in Supplement 1).^57^

### Covariates

We used Medicare beneficiary characteristics files to identify beneficiary age, sex, race and ethnicity (Hispanic, non-Hispanic Black, non-Hispanic White, and Other race or ethnicity [racial and ethnic groups other than Hispanic, non-Hispanic Black, non-Hispanic White]), county, zip code, original Medicare entitlement, dual eligibility, and death date. Race and ethnicity were based on Social Security Administration data, with a surname algorithm enhancing the identification of Hispanic and Asian origin.^58^ The number of comorbidities in the 12 months prior to the AWV index date was calculated using the Elixhauser Comorbidity Index.^59^ We also included 4 medical service utilization characteristics in the 12 months before the AWV index date: (1) number of hospitalizations (0, 1, 2, 3, or ≥4); (2) any neurologist visit; (3) any psychiatric visit; and (4) primary care provider (PCP) identified (eTable 1 in Supplement 1; Table 2).^60^ We used 2013 Rural-Urban Continuum Codes to distinguish metropolitan counties by population size and nonmetropolitan counties by degree of urbanization and adjacency to a metropolitan area.^61^ Based on previous studies,^5,18,61,62^ we extracted zip code–level high school graduation rates,^63^ county-level physical inactivity percentages,^64^ and county-level social association rates (Table 2).^62,64^

**Table 2.** Patient Characteristics Before and After Propensity Score Matching

Characteristic	Before propensity score matching			After propensity score matching		
	No. (%)		Standardized difference	No. (%)		Standardized difference
	No AWV (n = 483 083)	AWV (n = 66 433)		No AWV (n = 66 433)	AWV (n = 66 433)	
Comorbidities, No.						
0	171 907 (35.6)	12 301 (18.5)	−0.3915	12 298 (18.5)	12 301 (18.5)	0.0001
1	86 228 (17.8)	14 626 (22.0)	0.1044	15 002 (22.6)	14 626 (22.0)	−0.0136
2	80 482 (16.7)	14 405 (21.7)	0.1279	14 301 (21.5)	14 405 (21.7)	0.0038
≥3	144 466 (29.9)	25 101 (37.8)	0.1671	24 832 (37.4)	25 101 (37.8)	0.0084
Sex						
Female	251 372 (52.0)	38 560 (58.0)	−0.1210	38 421 (57.8)	38 560 (58.0)	−0.0042
Male	231 711 (48.0)	27 873 (42.0)	0.1210	28 012 (42.2)	27 873 (42.0)	0.0042
Age, y						
68-69	46 157 (9.6)	6112 (9.2)	−0.0122	5703 (8.6)	6112 (9.2)	0.0216
70-74	176 048 (36.4)	24 003 (36.1)	−0.0065	24 070 (36.2)	24 003 (36.1)	−0.0021
75-79	119 206 (24.7)	17 278 (26.0)	0.0306	17 390 (26.2)	17 278 (26.0)	−0.0038
80-84	76 739 (15.9)	10 583 (15.9)	0.0012	10 537 (15.9)	10 583 (15.9)	0.0019
≥85	64 933 (13.4)	8457 (12.7)	−0.0211	8733 (13.1)	8457 (12.7)	−0.0124
Race and ethnicity						
Hispanic	81 976 (17.0)	8396 (12.6)	−0.1222	7881 (11.9)	8396 (12.6)	0.0236
Non-Hispanic Black	32 591 (6.7)	3851 (5.8)	−0.0392	3287 (4.9)	3851 (5.8)	0.0377
Non-Hispanic White	352 802 (73.0)	51 792 (78.0)	0.1148	53 281 (80.2)	51 792 (78.0)	−0.0551
Other^a^	15 714 (3.3)	2394 (3.6)	0.0193	1984 (3.0)	2394 (3.6)	0.0346
Residential area at the county level						
Metropolitan	360 820 (74.7)	54 997 (82.8)	0.1988	55 302 (83.2)	54 997 (82.8)	−0.0122
Nonmetropolitan	122 263 (25.3)	11 436 (17.2)	−0.1988	11 131 (16.8)	11 436 (17.2)	0.0122
High school graduation rate at the county level in quantiles, %						
<80.8 (Quantile 1)	123 318 (25.5)	12 702 (19.1)	−0.1543	12 487 (18.8)	12 702 (19.1)	0.0083
80.8-88.0 (Quantile 2)	123 607 (25.6)	14 545 (21.9)	−0.0869	14 619 (22.0)	14 545 (21.9)	−0.0027
88.1-93.5 (Quantile 3)	118 184 (24.5)	18 249 (27.5)	0.0686	18 215 (27.4)	18 249 (27.5)	0.0011
>93.5 (Quantile 4)	117 974 (24.4)	20 937 (31.5)	0.1586	21 112 (31.8)	20 937 (31.5)	−0.0057
Original eligibility for Medicare						
Age 65 y	440 343 (91.2)	61 704 (92.9)	0.0638	62 594 (94.2)	61 704 (92.9)	−0.0546
Disabled or ESKD	42 740 (8.8)	4729 (7.1)	−0.0638	3839 (5.8)	4729 (7.1)	0.0546
Dual eligibility						
No	438 883 (90.9)	61 431 (92.5)	0.0586	62 160 (93.6)	61 431 (92.5)	−0.0431
Yes	44 200 (9.1)	5002 (7.5)	−0.0586	4273 (6.4)	5002 (7.5)	0.0431
Physical inactivity at the county level in quantiles, %						
<22.7 (Quantile 1)	92 545 (19.2)	14 184 (21.4)	0.0546	14 516 (21.9)	14 184 (21.4)	−0.0121
22.7-24.2 (Quantile 2)	145 717 (30.2)	21 430 (32.3)	0.0452	20 894 (31.5)	21 430 (32.3)	0.0173
24.3-27.5 (Quantile 3)	115 199 (23.8)	15 081 (22.7)	−0.0271	15 342 (23.1)	15 081 (22.7)	−0.0094
>27.5 (Quantile 4)	129 622 (26.8)	15 738 (23.7)	−0.0724	15 681 (23.6)	15 738 (23.7)	0.0020
Social association rate by 10 000 population at the county level in quantiles						
<5.7 (Quantile 1)	133 154 (27.6)	17 511 (26.4)	−0.0271	17 170 (25.8)	17 511 (26.4)	0.0117
5.7-7.3 (Quantile 2)	103 162 (21.4)	16 768 (25.2)	0.0920	16 870 (25.4)	16 768 (25.2)	−0.0035
7.4-10.0 (Quantile 3)	118 837 (24.6)	16 440 (24.7)	0.0034	16 623 (25.0)	16 440 (24.7)	−0.0064
>10 .0 (Quantile 4)	127 930 (26.5)	15 714 (23.7)	−0.0653	15 770 (23.7)	15 714 (23.7)	−0.0020
Having a primary care provider 12 mo before the AWV index date						
No	295 979 (61.3)	28 080 (42.3)	−0.3873	29 136 (43.9)	28 080 (42.3)	−0.0321
Yes	187 104 (38.7)	38 353 (57.7)	0.3873	37 297 (56.1)	38 353 (57.7)	0.0321
Having at least 1 neurologist visit 12 mo before the AWV index date						
No	457 578 (94.7)	61 783 (93.0)	−0.0717	62 514 (94.1)	61 783 (93.0)	−0.0448
Yes	25 505 (5.3)	4650 (7.0)	0.0717	3919 (5.9)	4650 (7.0)	0.0448
Having at least 1 psychiatric visit 12 mo before the AWV index date						
No	478 275 (99.0)	65 498 (98.6)	−0.0378	65 736 (99.0)	65 498 (98.6)	−0.0325
Yes	4808 (1.0)	935 (1.4)	0.0378	697 (1.0)	935 (1.4)	0.0325
Hospitalizations 12 mo before the AWV index date, No.						
0	423 618 (87.7)	57 361 (86.3)	−0.0401	57 891 (87.1)	57 361 (86.3)	−0.0235
1	44 535 (9.2)	6890 (10.4)	0.0388	6629 (10.0)	6890 (10.4)	0.0130
2	10 065 (2.1)	1487 (2.2)	0.0106	1321 (2.0)	1487 (2.2)	0.0174
3	3036 (0.6)	441 (0.7)	0.0044	386 (0.6)	441 (0.7)	0.0105
≥4	1829 (0.4)	254 (0.4)	0.0006	206 (0.3)	254 (0.4)	0.0123

Abbreviations: AWV, annual wellness visit; ESKD, end-stage kidney disease.

^a^ Other includes racial and ethnic groups other than Hispanic, non-Hispanic Black, and non-Hispanic White.

### Statistical Analysis

To control for differences between beneficiaries with and beneficiaries without an AWV, we did propensity score matching (a 1:1 match). The propensity score of recipients of an AWV was generated using a logistic regression model that includes the covariates listed in Table 2. The AWV month was an exact match in the propensity score method. For each beneficiary with an AWV, we performed greedy-nearest-neighbor matching to select 1 beneficiary without an AWV within a caliper equal to 0.2 SDs of the logit of the propensity score. Standardized differences were calculated to assess the balance of covariates between groups with and groups without an AWV before and after propensity score matching. A standardized difference of less than 0.10 between groups indicated a balance in covariates.

Our main analysis was based on the 2018 AWV regardless of any AWVs during the follow-up period. Cumulative incidence function plots were used to show the probability of each outcome (MCI or ADRD, MCI, ADRD) by group. We constructed a conditional Fine-Gray competing risk model to evaluate the association of AWV receipt in 2018 with the first MCI or ADRD diagnosis during follow-up in 3 ways: MCI or ADRD, MCI, and ADRD, demonstrated as hazard ratio (HR) and 95% CI.

We performed 2 sensitivity analyses. In the first, we further censored the no-AWV cohort when they initiated their first AWV and the AWV cohort if they had no continuing AWV within 15 months of the previous AWV. A conditional Fine-Gray competing risk model was constructed, and HRs with 95% CIs were reported.

In the second sensitivity analysis, we treated AWV as a time-dependent variable. Annual wellness visit exposure status could transfer to no-AWV status, and no-AWV status could move to AWV exposure. We constructed an unconditional Fine-Gray competing risk model adjusted for all covariables to evaluate the association of AWV status with first MCI or ADRD diagnosis in 3 ways, as for the main analysis. All statistical analyses were performed using SAS/STAT software, version 9.4 (SAS Institute Inc). All *P* values were from 2-sided tests and results were deemed statistically significant at *P* < .05.

## Results

### Demographic Characteristics

Among 549 516 Medicare beneficiaries aged 68 years or older with no diagnosis of MCI or ADRD before 2018 (mean [SD] age, 76.7 [6.6] years; 289 932 women [52.8%] and 259 584 men [47.2%]; 90 372 Hispanic beneficiaries [16.4%], 36 442 non-Hispanic Black beneficiaries [6.6%], 404 594 non-Hispanic White beneficiaries [73.6%], and 18 108 beneficiaries of Other race or ethnicity [3.3%]), 66 433 (12.1%) had an incident AWV in 2018. Beneficiaries receiving an AWV were more likely than those who did not receive an AWV to be female, be non-Hispanic White, have a higher educational level, reside in a metropolitan area, have more comorbidities, and have an identified PCP in the 12 months before the AWV index date. After propensity score matching, the characteristics were balanced between the 2 groups, with a standardized difference of less than 10% (Table 2).

### Main Analysis

As shown in Figure, A, the probabilities of developing MCI or ADRD were 5.2% at the 1-year follow-up, 9.8% at the 2-year follow-up, and 22.8% at the 5-year follow-up for the AWV group and 4.5% at the 1-year follow-up, 8.9% at the 2-year follow-up, and 21.3% at the 5-year follow-up for the no-AWV group. Receipt of AWV was associated with an 8% increase in first MCI or ADRD diagnosis (HR, 1.08 [95% CI, 1.05-1.10]) (Table 3).

**Figure.**
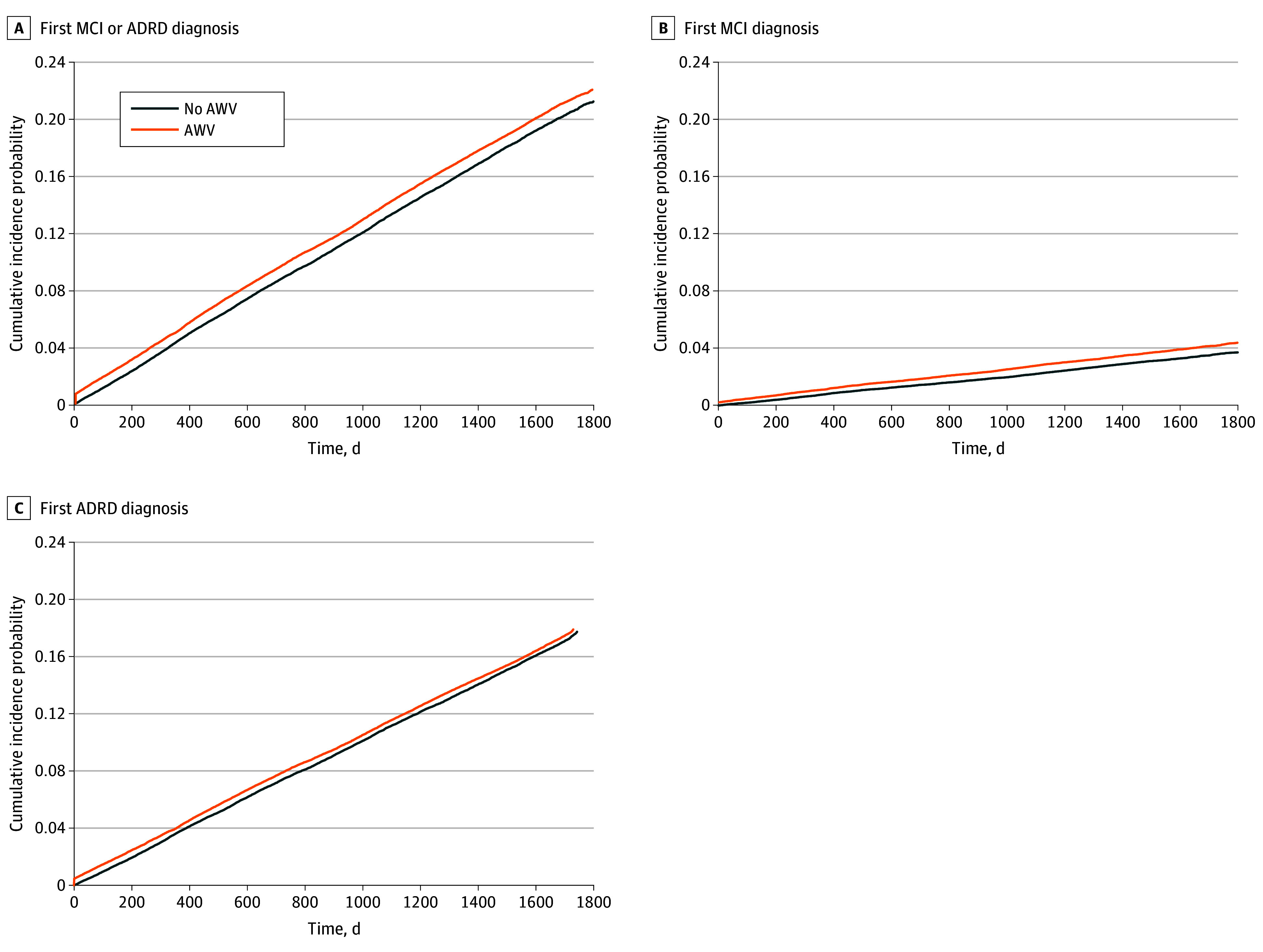
Cumulative Incidence Function Plots for the First Diagnosis of Mild Cognitive Impairment (MCI) or Alzheimer Disease and Related Dementias (ADRD) in 3 Ways: MCI or ADRD, MCI, and ADRD A. The cumulative incidence function plot for the first MCI or ADRD diagnosis. B, The cumulative incidence function plot for the first MCI diagnosis. C, The cumulative incidence function plot for the first ADRD diagnosis. A total of 321 patients had the same date for the annual wellness visit (AWV) and receiving an MCI or ADRD diagnosis, meaning that the patient was diagnosed as having MCI or ADRD on the same day the AWV visit was delivered. As shown in panels B and C, AWV recipients had a timelier first diagnosis of MCI, but timing of first diagnosis of ADRD differed little.

**Table 3.** Main Models and 2 Sensitivity Analyses: Conditional Fine-Gray Competing Risk Models for the MCI or ADRD Diagnosis (Outcome) in 3 Ways (MCI or ADRD, MCI Only, and ADRD Only)

AWV exposure or MCI or ADRD diagnosis	Hazard ratio (95% CI)		
	Main model	First sensitivity analysis	Second sensitivity analysis
AWV: MCI or ADRD, No.			
0	1 [Reference]	1 [Reference]	1 [Reference]
1	1.08 (1.05-1.10)	1.09 (1.06-1.12)	1.06 (1.04-1.09)
AWV: MCI only, No.			
0	1 [Reference]	1 [Reference]	1 [Reference]
1	1.21 (1.16-1.27)	1.36 (1.28-1.45)	1.40 (1.32-1.49)
AWV: ADRD only, No.			
0	1 [Reference]	1 [Reference]	1 [Reference]
1	1.04 (1.02-1.06)	1.03 (1.00-1.06)	1.00 (0.97-1.03)

Abbreviations: ADRD, Alzheimer disease and related dementias; AWV, annual wellness visit; MCI, mild cognitive impairment.

The AWV group had a higher proportion of MCI among beneficiaries developing MCI or ADRD during the follow-up period compared with the no-AWV group. The probability of developing MCI was 1.1% at the 1-year follow-up, 1.9% at the 2-year follow-up, and 4.4% at the 5-year follow-up for the AWV group and 0.8% at the 1-year follow-up, 1.5% at the 2-year follow-up, and 3.7% at the 5-year follow-up for the no-AWV group. The median time from first AWV to first MCI diagnosis was 692 days (IQR, 269-1132 days); the median time to first MCI diagnosis from the index date for the no-AWV group was 768 days (IQR, 360-1199 days), a 76-day difference (Figure, B). The probability of developing ADRD was 4.1% at the 1-year follow-up, 7.9% at the 2-year follow-up, and 18.6% at the 5-year follow-up for the AWV group and 3.8% at the 1-year follow-up, 7.5% at the 2-year follow-up, and 17.7% at the 5-year follow-up for the no-AWV group. The median time to first diagnosis of ADRD was 712 days (IQR, 332-1154 days) for the AWV group and 752 days (IQR, 365-1175 days) for the no-AWV group, a 40-day difference (Figure, C). Receipt of AWV was associated with a 21% increase in MCI diagnosis (HR, 1.21 [95% CI, 1.16-1.27]) and a 4% increase in ADRD diagnosis (HR, 1.04 [95% CI, 1.02-1.06]) (Table 3).

### Sensitivity Analyses

In the follow-up period, 17.9% of beneficiaries in the AWV group and 16.9% of beneficiaries in the no-AWV group lost Medicare coverage; 8.5% of beneficiaries in the AWV group and 10.5% of beneficiaries in the no-AWV group died. In addition, from 2019 to 2022, among the AWV group, 25.5% of beneficiaries received no AWVs, 22.8% of beneficiaries received 1 AWV, 20.9% of beneficiaries received 2 AWVs, and 30.8% of beneficiaries received 3 or more AWVs; among the no-AWV group, 35.4% of beneficiaries received at least 1 AWV from 2019 to 2022.

As shown in Table 3, our first sensitivity analysis, censoring AWV in the follow-up period, showed a stronger association of AWV with MCI (HR, 1.36 [95% CI, 1.28-1.45]). The second sensitivity analysis, treating AWV as a time-dependent variable and adjusting for covariates, also showed a higher association of AWV with the first MCI diagnosis (HR, 1.40 [95% CI, 1.32-1.49]), but a nonsignificant association of AWV with first ADRD diagnosis (HR, 1.00 [95% CI, 0.97-1.03]).

Beneficiaries with any hospitalizations 12 months before the AWV index date were more likely to receive a diagnosis of ADRD, with inconclusive findings for MCI. Beneficiaries who had a PCP 12 months before the AWV index date were more likely to receive a diagnosis of MCI, but the association was nonsignificant for ADRD. Beneficiaries who had at least 1 neurologist visit or at least 1 psychiatric visit 12 months before the AWV index date were more likely to receive a diagnosis of both MCI and ADRD (eTable 2 in Supplement 1).

## Discussion

This cohort study assessed the association of incident AWV in 2018 with the first ADRD or MCI diagnosis from the AWV index date through the end of 2022 in older adults with Medicare fee-for-service benefits in Texas. We found that AWV receipt was associated with a 21% increase in MCI (HR, 1.21 [95% CI, 1.16-1.27]) and a 4% increase in ADRD diagnosis (HR, 1.04 [95% CI, 1.02-1.06]). In other words, AWV recipients had a higher rate of first MCI diagnosis, with little difference in first ADRD diagnosis. Results of the main analysis and 2 sensitivity analyses were consistent with our hypothesis that receiving an AWV would be associated with increased MCI diagnosis and minimum difference in first ADRD diagnosis. To our knowledge, our cohort study is the first to show the association of AWVs with early recognition of MCI in older adults.

In 2020, the US Preventive Services Task Force found insufficient evidence that cognitive impairment screening improves outcomes for older adult patients or their family caregivers,^40,65^ mainly because established pharmacologic agents did not alter the disease progression of cognitive impairment.^16^ Since 2023, 2 pharmacologic agents approved by the US Food and Drug Administration, lecanemab^66^ and donanemab,^67^ that target the underlying Alzheimer disease process of amyloid plaque formation in patients’ brains have been showm to significantly slowed clinical progression over 18 months and 76 weeks, respectively, in patients with early symptomatic Alzheimer disease and amyloid and tau pathology. Screening for cognitive impairment could improve patient outcomes through timely initiation of dementia care and medications such as lecanemab and donanemab.^61,66^

Community-dwelling older adults with MCI are more likely to experience a repeat fracture, leading to rehospitalization.^68^ Three consecutive years of AWVs were found to lower the risk of falls.^30^ The benefits of early recognition and diagnosis of cognitive impairment are numerous; they allow clinicians to develop patient-centered care and strategies (eg, reducing exposure to potentially inappropriate medications,^19^ acute care use,^20,21,22,23^ and fall-related injuries^24,25,26,27,28,29,30^) and medical care utilization (eg, maximizing independent living and initiating early advance care planning conversations).^69^

We also found that patients in the AWV group had a timelier first MCI diagnosis and a timelier first ADRD diagnosis than those in the no-AWV group. A 76-day difference in first MCI diagnosis may appear clinically insignificant, but has clinical patient care implications that matter. In clinically complex older patients with multiple morbidities, this almost 11-week period can be the difference in recognizing and preventing medication nonadherence or medication errors, possibly mitigating unplanned emergency department visits for medication toxicity or disease exacerbation.^41,42^ However, severe impairment might be more easily recognized by both nonclinical and clinical health care professionals as well as caregivers or family members, consistent with our findings that the AWV group had a 40-day earlier ADRD diagnosis than the no-AWV group.

Having a PCP before the AWV index date was associated with a significant increase in first MCI diagnosis, but not first ADRD diagnosis. A recent study found that among clinicians with at least 25 attributed patients and more than 50% of patients with an MCI or ADRD diagnosis, those practicing geriatric medicine had a mean MCI detection rate 2.5- to 3.7-fold that of other types of PCPs.^33^ Studies should examine the differences in PCP attitudes toward AWVs and cognition assessment across practice patterns (eg, solo vs team practice). Having at least 1 neurologist or psychiatrist visit 12 months before the AWV index date was associated with an increase in first MCI or ADRD diagnosis. One study found that only 47.3% of adults with self-reported cognitive decline had discussed their confusion or memory loss with a health care professional.^70^ Patients concerned about memory and cognitive decline should consult a health care professional to identify causes.^61^ Research is needed to identify factors associated with delay in confirming MCI or ADRD diagnosis, such as access-related barriers and known risk factors in which PCPs may intervene (eg, coordinating referrals to a neurologist, psychiatrist, or social service).^61^

Our study follow-up period for MCI or ADRD diagnosis extended through the period of the COVID-19 pandemic. Therefore, to understand the potential effect of the COVID-19 pandemic and related mitigation measures, we restricted our follow-up period to the end of 2019 (eTable 3 in Supplement 1). Compared with the findings in Table 3, we found that the magnitude of association of AWV with MCI or ADRD diagnosis was stronger in the pre–COVID-19 follow-up period, suggesting the possibility of reduced clinic visits during the most challenging phase of the COVID-19 pandemic. In this scenario, such reductions in patient visits to health care professionals may affect MCI recognition or even dementia diagnosis. However, we cannot confirm that the COVID-19 pandemic caused the lower association observed through follow-up to 2022. Further study will help clarify the association of COVID-19 with early diagnosis of dementia.

### Future Research Directions

Future studies should analyze additional patient- and caregiver-level information to better understand potential patient self-selection bias (ie, caregivers’ preference for taking patients with MCI for AWVs). Studies should also determine the roles of physicians and advanced practice clinicians (ie, nurse practitioners or physician assistants) in AWV delivery. Results can guide the development and implementation of AWV protocols to support early recognition of cognitive impairment.

### Limitations

This study has some limitations. Annual wellness visits have a patient self-selection bias that administrative data cannot capture. For example, the preference for an AWV is unmeasured. Caregivers might be more likely to take patients with MCI for AWVs. Including AWV preference in national surveys linked to Medicare data could address this unmeasured confounding. Another limitation is the limited generalizability to individuals outside of Texas fee-for-service beneficiaries; Texas has a high number of Black and Hispanic individuals with dementia^43,44,45,46,47^ and a low use of AWVs.^34,53^ Also, analyses were limited to Medicare billing data, which do not capture how health care professionals perform AWVs or clinics’ efforts to facilitate timely referrals to neurologists or psychiatrists for further cognitive impairment evaluation. We had no patient-level information on education, marital status, living arrangements, physical activity, and social life, posing challenges to interpreting findings via the lens of social determinants of health.

## Conclusions

This cohort study revealed that AWV recipients had a higher rate of first MCI diagnosis (21%) than those who did not receive an AWV, but little difference in first ADRD diagnosis (4%). Our study is the first showing the association of AWVs with early recognition of MCI in older adults. Annual wellness visits represent 1 strategy to address the family care burden of older adults with cognitive impairment by providing timely dementia care^1,2,3,4,5^ to maximize independent living for older adults with cognitive impairment.^6,7,8,9,10,11,12,13,14^ The Centers for Medicare & Medicaid Services should publicize the benefits of AWVs, including the potential to preserve independent living and aging in place of community-dwelling older adults through timely screening, dementia care, and treatment.

## Notes

Supplement 1. **eTable 1.** Summary of the Sources and Definition of Study Variables From the *International Statistical Classification of Diseases and Related Health Problems,* 10th Revision, Clinical Modification (*ICD-10-CM*) **eTable 2.** The Second Sensitivity Analysis: Full Conditional Fine-Gray Competing Risk Models for the MCI/ADRD Diagnosis (Outcome) in 3 Ways (MCI/ADRD, MCI, ADRD) **eTable 3.** The Main Models and the First Sensitivity Analyses in the Pre-COVID Follow-Up Period (Up to December 2019): Conditional Fine-Gray Competing Risk Models for the MCI/ADRD Diagnosis (Outcome) in 3 Ways (MCI/ADRD, MCI Only, ADRD Only)

Supplement 2.
Data Sharing Statement
