# Supplementary material for: Annual Wellness Visits and Early Dementia Diagnosis Among Medicare Beneficiaries
Source: JAMA Netw Open. 2024 Oct 8;7(10):e2437247. doi: 10.1001/jamanetworkopen.2024.37247 (PMC11581498; doi:10.1001/jamanetworkopen.2024.37247)
Supplement: Supplement 1. — eTable 1. Summary of the Sources and Definition of Study Variables From the International Statistical Classification of Diseases and Related Health Problems, 10th Revision, Clinical Modification (ICD-10-CM) eTable 2. The Second Sensitivity Analysis: Full Conditional Fine-Gray Competing Risk Models for the MCI/ADRD Diagnosis (Outcome) in 3 Ways (MCI/ADRD, MCI, ADRD) eTable 3. The Main Models and the First Sensitivity Analyses in the Pre-COVID Follow-Up Period (Up to December 2019): Conditional Fine-Gray Competing Risk Models for the MCI/ADRD Diagnosis (Outcome) in 3 Ways (MCI/ADRD, MCI Only, ADRD Only) [file jamanetwopen-e2437247-s001.pdf]

Supplementary Online Content

Tzeng HM, Raji MA, Shan Y, Cram P, Kuo YF. Annual wellness visits and early dementia diagnosis among Medicare beneficiaries. *JAMA Netw Open.* 2024;7(10):e2437247. doi:10.1001/jamanetworkopen.2024.37247

eTable 1. Summary of the Sources and Definition of Study Variables From the *International Statistical Classification of Diseases and Related Health Problems,* 10th Revision, Clinical Modification (*ICD-10-CM*)
eTable 2. The Second Sensitivity Analysis: Full Conditional Fine-Gray Competing Risk Models for the MCI/ADRD Diagnosis (Outcome) in 3 Ways (MCI/ADRD, MCI, ADRD)
eTable 3. The Main Models and the First Sensitivity Analyses in the Pre-COVID Follow-Up Period (Up to December 2019): Conditional Fine-Gray Competing Risk Models for the MCI/ADRD Diagnosis (Outcome) in 3 Ways (MCI/ADRD, MCI only, ADRD only)

This supplementary material has been provided by the authors to give readers additional information about their work.

© 2024 Tzeng HM et al. *JAMA Network Open.*

eTable 1. Summary of the Sources and Definition of Study Variables From the *International Statistical Classification of Diseases and Related Health Problems*, 10th Revision, Clinical Modification (*ICD-10-CM*)

Variable	Data Source	Definition
Annual wellness visit (AWV)	Outpatient Standard Analytical files, Carrier file	HCPCS: G0438 (an initial AWV), G0439 (a subsequent AWV)[a]
The number of neurologist visits (yes as having any number of neurologist visits/no)	MedPAR<br>Outpatient Standard Analytical files, Carrier file	E&M billing codes: 99201-99205, 99211-99215, 99241-99245 with specialty codes 13, 86
The number of psychiatrists visits (yes as having any number of psychiatrist visits/no)	MedPAR<br>Outpatient Standard Analytical files, Carrier file	E&M billing codes: 99201-99205, 99211-99215, 99241-99245 with specialty codes 26, 27
Having a primary care provider (PCP) (yes/no)	MedPAR<br>Outpatient Standard Analytical files, Carrier file	Having a PCP with the credential as family medicine, internal medicine, general practice, geriatrics physician, nurse practitioner, or physician assistant who filed ≥2 outpatient E&M billing codes: 99201–99205,

		99211–99215
Cognitive impairment: Mild cognitive impairment (MCI) vs. Alzheimer's disease and related dementias (ADRD)	MedPAR<br>Outpatient Standard Analytical files, Carrier file	MCI: ICD-9 311.83, ICD-10 G31.84.<br>ADRD, see the Chronic Condition Warehouse (CCW) ADRD algorithm specification below.
**CCW ADRD Algorithm Specification**		
ADRD	**ICD-9-CM**<br>331.0, 331.11, 331.19, 331.2, 331.7, 290.0, 290.10, 290.11, 290.12, 290.13, 290.20, 290.21, 290.3, 290.40, 290.41, 290.42, 290.43, 294.0, 294.10, 294.11, 294.20, 294.21, 294.8, 797	**ICD-10-CM**<br>F01.50, F01.51, F02.80, F02.81, F03.90, F03.91, F04, G13.8, F05, F06.1, F06.8, G30.0, G30.1, G30.8, G30.9, G31.1, G31.2, G31.01, G31.09, G94, R41.81, R54

E&M = Evaluation and Management

HCPCS = Healthcare Common Procedure Coding System

MedPAR = Medicare Provider Analysis and Review

[a]: Centers for Medicare and Medicaid Services Medicare Learning Network. Medicare wellness visits: Medicare physical exams coverage. https://www.cms.gov/Outreach-and-Education/Medicare-Learning-Network-MLN/MLNProducts/preventive-services/medicare-wellness-visits.html. Published 2024. Accessed March 31, 2024.

© 2024 Tzeng HM et al. *JAMA Network Open.*

eTable 2. The Second Sensitivity Analysis: Full Conditional Fine-Gray Competing Risk Models for the MCI/ADRD Diagnosis (Outcome) in 3 ways (MCI/ADRD, MCI, ADRD)

**MCI/ADRD diagnosis /Characteristic**	**MCI/ADRD**	**MCI ONLY**	**ADRD ONLY**
**AWV**			
0	REF	REF	REF
1	1.06 (1.04, 1.09)	1.40 (1.32, 1.49)	1.00 (0.97, 1.03)
**Comorbidities**			
0	REF	REF	REF
1	1.12 (1.07, 1.18)	1.17 (1.05, 1.30)	1.11 (1.05, 1.17)
2	1.23 (1.17, 1.29)	1.27 (1.14, 1.42)	1.22 (1.15, 1.28)
3+	1.54 (1.47, 1.61)	1.40 (1.26, 1.56)	1.57 (1.49, 1.65)
**Sex**			
Female	REF	REF	REF
Male	0.94 (0.91, 0.96)	0.93 (0.87, 0.99)	0.94 (0.91, 0.97)
**Age at diagnosis**			
68–69 Years	REF	REF	REF
70–74 Years	1.30 (1.22, 1.40)	1.19 (1.04, 1.36)	1.34 (1.24, 1.45)
75–79 Years	2.18 (2.04, 2.34)	1.64 (1.43, 1.88)	2.38 (2.20, 2.57)
80–84 Years	3.48 (3.25, 3.73)	2.09 (1.81, 2.42)	3.99 (3.68, 4.32)
≥85 Years	6.11 (5.70, 6.55)	2.47 (2.13, 2.85)	7.45 (6.89, 8.07)

© 2024 Tzeng HM et al. *JAMA Network Open.*

MCI/ADRD diagnosis /Characteristic	MCI/ADRD	MCI ONLY	ADRD ONLY
**Race and ethnicity**			
Non-Hispanic White	REF	REF	REF
Hispanic	0.96 (0.92, 1.01)	0.83 (0.74, 0.93)	0.99 (0.94, 1.04)
Non-Hispanic Black	1.06 (1.00, 1.12)	0.97 (0.84, 1.12)	1.08 (1.02, 1.15)
Other	0.89 (0.82, 0.97)	0.84 (0.70, 1.01)	0.90 (0.82, 0.99)
**Residential area at the county level**			
Metropolitan	REF	REF	REF
Non-metropolitan	1.02 (0.98, 1.07)	0.93 (0.83, 1.03)	1.04 (1.00, 1.09)
**High school graduation rate at the county level in quantiles**			
<80.8% (1)	REF	REF	REF
80.8–88.0% (2)	1.00 (0.96, 1.04)	1.10 (0.99, 1.22)	0.98 (0.94, 1.03)
88.1–93.5% (3)	1.00 (0.96, 1.04)	1.04 (0.94, 1.16)	1.00 (0.95, 1.04)
>93.5% (4)	0.99 (0.95, 1.03)	1.23 (1.12, 1.36)	0.94 (0.90, 0.98)
**Original entitlement**			
Aged	REF	REF	REF
Disabled or ESRD	1.46 (1.39, 1.54)	1.12 (0.98, 1.28)	1.55 (1.47, 1.64)
**Dual eligibility**			
No	REF	REF	REF
Yes	1.44 (1.37, 1.51)	1.01 (0.88, 1.17)	1.51 (1.43, 1.59)

© 2024 Tzeng HM et al. *JAMA Network Open.*

MCI/ADRD diagnosis /Characteristic	MCI/ADRD	MCI ONLY	ADRD ONLY
**Physical inactivity percentage at the county level in quantiles**			
<22.7% (1)	REF	REF	REF
22.7–24.2% (2)	0.99 (0.95, 1.02)	1.02 (0.93, 1.10)	0.98 (0.94, 1.03)
24.3–27.5% (3)	0.93 (0.89, 0.97)	0.86 (0.77, 0.95)	0.95 (0.90, 1.00)
>27.5% (4)	0.97 (0.92, 1.01)	0.78 (0.69, 0.87)	1.01 (0.96, 1.06)
**Social association rate by 10,000 population at the county level in quantiles**			
<5.7 (1)	REF	REF	REF
5.7–7.3 (2)	1.00 (0.96, 1.04)	1.17 (1.07, 1.27)	0.96 (0.92, 1.01)
7.4–10 (3)	1.00 (0.95, 1.04)	1.08 (0.97, 1.20)	0.98 (0.93, 1.03)
>10 (4)	0.97 (0.92, 1.02)	0.99 (0.87, 1.11)	0.97 (0.91, 1.02)
**Having a primary care provider 12 months before the AWV index date**			
No	REF	REF	REF
Yes	1.03 (1.00, 1.06)	1.17 (1.10, 1.25)	1.00 (0.97, 1.03)
**Having at least one neurologist visit 12 months before the AWV index date**			
No	REF	REF	REF
Yes	1.66 (1.59, 1.73)	2.17 (1.99, 2.37)	1.55 (1.47, 1.63)
**Having at least one psychiatric visit 12 months before the AWV index date**			
No	REF	REF	REF
Yes	1.73 (1.57, 1.91)	2.11 (1.75, 2.54)	1.62 (1.45, 1.82)

© 2024 Tzeng HM et al. *JAMA Network Open.*

MCI/ADRD diagnosis /Characteristic	MCI/ADRD	MCI ONLY	ADRD ONLY
**Number of hospitalizations 12 months before the AWV index date**			
0	REF	REF	REF
1	1.19 (1.15, 1.24)	1.10 (1.00, 1.21)	1.21 (1.16, 1.26)
2	1.33 (1.23, 1.43)	1.03 (0.84, 1.26)	1.39 (1.28, 1.51)
3	1.53 (1.34, 1.74)	1.12 (0.78, 1.59)	1.63 (1.41, 1.87)
4+	1.96 (1.66, 2.31)	1.31 (0.82, 2.09)	2.10 (1.76, 2.49)

ADRD = Alzheimer's Disease and Related Dementias

AWV = annual wellness visit

CI = confidence interval

ESRD = end-stage renal disease

HR = hazard ratio

MCI = mild cognitive impairment

REF = the reference group

Notes:

For the second sensitivity analysis, we treated AWV as a time-dependent variable. The AWV status can be transferred to a non-AWV status, and the non-AWV status can be transferred to an AWV status. We constructed the model adjusted for all covariables to evaluate the association of AWV status on each outcome. We treated death as a competing event.

© 2024 Tzeng HM et al. *JAMA Network Open.*

eTable 3. The Main Models and the First Sensitivity Analyses in the Pre-COVID Follow-Up Period (Up to December 2019): Conditional Fine-Gray Competing Risk Models for the MCI/ADRD Diagnosis (Outcome) in 3 Ways (MCI/ADRD, MCI only, ADRD only)

**Characteristic:**	**The main models**	**The 1st sensitivity analysis**	**The 2nd sensitivity analysis**
**AWV exposure /The MCI/ADRD diagnosis**			
AWV: MCI/ADRD	HR (95% CI)	HR (95% CI)	HR (95% CI)
0	REF	REF	REF
1	1.14 (1.11, 1.18)	1.17 (1.13, 1.21)	1.12 (1.07, 1.16)
AWV: MCI ONLY			
0	REF	REF	REF
1	1.36 (1.27, 1.46)	1.45 (1.35, 1.57)	1.41 (1.29, 1.55)
AWV: ADRD ONLY			
0	REF	REF	REF
1	1.09 (1.06, 1.13)	1.11 (1.07, 1.15)	1.06 (1.01, 1.11)

ADRD = Alzheimer's Disease and Related Dementias

AWV = annual wellness visit

© 2024 Tzeng HM et al. *JAMA Network Open.*

CI = confidence interval

HR = hazard ratio

MCI = mild cognitive impairment

REF = the reference group

Notes.

The main model: We evaluated the association of AWV receipt in 2018 on each outcome during the pre-COVID follow-up (up to December 2019). This analysis was based on the 2018 AWV regardless of whether patients continued having AWV or initiated AWV during follow-up. All patients are censored at lost coverage or at the end of the study. We treated death as a competing event.

The 1st sensitivity analysis: We censored the non-AWV cohort when they initiated their first AWV visit and the AWV cohort if they had no continuing AWV visit after 15 months of the first AWV visit. We treated death as a competing event.

The 2nd sensitivity analysis: We treated AWV as a time-dependent variable. The AWV status can be transferred to a non-AWV status, and the non-AWV status can be transferred to an AWV status. We constructed the model adjusted for all covariables to evaluate the association of AWV status on each outcome. We treated death as a competing event.

© 2024 Tzeng HM et al. *JAMA Network Open.*
