# Supplementary material for: Annual Wellness Visits and Early Dementia Diagnosis Among Medicare Beneficiaries
Source: JAMA Netw Open. 2024 Oct 8;7(10):e2437247. doi: 10.1001/jamanetworkopen.2024.37247 (PMC11581498; doi:10.1001/jamanetworkopen.2024.37247)
Supplement: Supplement 2. — Data Sharing Statement [file jamanetwopen-e2437247-s002.pdf]

Data Sharing Statement

Tzeng. Annual Wellness Visits and Early Dementia Diagnosis Among Medicare Beneficiaries. *JAMA Netw Open*. Published October 08, 2024. doi:10.1001/jamanetworkopen.2024.37247

Data
Data available: No

Additional Information
Explanation for why data not available: We could not share the data used for this study due to the Data User Agreement (DUA) policies set by the Centers for Medicare & Medicaid.
